# K-ras mutation in the endometrium of tamoxifen-treated breast cancer patients, with a comparison of tamoxifen and toremifene

**DOI:** 10.1038/sj.bjc.6602456

**Published:** 2005-03-08

**Authors:** T Hachisuga, H Tsujioka, S Horiuchi, T Udou, M Emoto, T Kawarabayashi

**Affiliations:** 1 Department of Obstetrics and Gynecology, the School of Medicine, Fukuoka University, 45-1, 7-chome, Nanakuma, Jonan-ku, Fukuoka 814-0180, Japan

**Keywords:** tamoxifen, toremifene, K-ras, endometrium, menstruation

## Abstract

The putative presence of a mutation in codon 12 of the K-ras gene was investigated in the endometrium of tamoxifen (TAM) and toremifene (TOR)-treated breast cancer patients. DNA was extracted from fresh cytologic samples of the endometrium in 86 TAM and 21 TOR-treated breast cancer patients. Mutations were detected by enriched PCR and an enzyme-linked mini-sequence assay (ELMA). K-ras mutation was found in 35 TAM-treated endometrial samples, and in only one TOR-treated endometrium (*P*<0.003). In 24 premenopausal patients, K-ras mutation was found in seven (43.8%) of 16 patients with less than 47 months of TAM treatment, while none was found in eight patients with more than 48 months of TAM treatment (*P*<0.03). In 62 postmenopausal-amenorrheic patients, K-ras mutation was found in three (15.8%) of 19 patients with less than 23 months of TAM treatment, while it was found in 16 (61.5%) of 26 patients with 24–47 months of TAM treatment and nine (52.9%) of 17 patients with more than 48 months of TAM treatment (*P*=0.002). The presence of K-ras mutation is significantly influenced by the duration of TAM treatment and menstrual status of the patients. TOR may have a lower potential genotoxicity than TAM.

Tamoxifen (TAM) is a nonsteroidal triphenylethylene derivative that has proven to be effective in the adjuvant treatment of breast cancer by increasing both the disease-free period and overall survival rate. Tamoxifen is generally well tolerated and had been believed to have little side effects, but a number of large epidemiological studies (Fisher *et al*, 1994; Rutqvist *et al*, 1995; Bernstein *et al*, 1999; Bergman *et al*, 2000) have confirmed that the relative risk of endometrial cancers is estimated to be two- to three-fold of control, and the risk increases with both the duration and cumulative dose of TAM treatment. Tamoxifen exhibits the agonistic and antagonistic effects of oestrogen in different tissues, depending on the ambient oestradiol concentration (Mourits *et al*, 2001). Several investigators have suggested the oestrogenic effect of TAM on the development of endometrial cancer (Bernstein *et al*, 1999; Deligdisch *et al*, 2000).

On the other hand, TAM-DNA adducts are detected in a half of the endometrial samples obtained from TAM-treated women (Shibutani *et al*, 2000a). The site-specific dG-N^2^-TAM adducts display a high miscoding and mutagenic potential and primarily generate G to T transversions in mammalian cells (Terashima *et al*, 1999). One study has concluded that if TAM-DNA adducts are not repaired, then the potential risk of developing endometrial cancer in the TAM-treated women may be increased (Shibutani *et al*, 2000b). Toremifene (TOR), a chlorinated TAM derivative, has been also used for adjuvant hormonal treatment in breast cancer. Both TAM and TOR have a similar oestrogenic activity in endometrial cancer cells (O’Regan *et al*, 1998) and produce a similar increase in the endometrial thickness of postmenopausal breast cancer patients (Tomas *et al*, 1995). However, TOR has been reported to show a lower genotoxicity than TAM (Shibutani *et al*, 2001).

In the ras gene superfamily, the codon 12 (-TGGTG-) of the K-ras gene is the most frequently mutated codon in human cancers. K-ras mutations are considered to correlate with the phenotypic progression from atypical hyperplasia to endometrial cancer. A mutation in codon 12 of the K-ras gene has been identified in a range from 4.5 to 23% of atypical endometrial hyperplasia cases (Lagarda *et al*, 2001; Sun *et al*, 2002) and in from 11 to 26% of endometrioid carcinomas of the endometrium (Semczuk *et al*, 1998; Lax *et al*, 2000). The codons 12 and 14 of the K-ras gene are reported to be hotspots for carcinogen-DNA adduct formation in human bronchial epithelial cells (Feng *et al*, 2002). The DNA adducts that formed at codon 12 of the K-ras gene were poorly repaired in comparison to those at other codons, including codon 14 (Feng *et al*, 2002). A high incidence of mutations in codon 12 of the K-ras gene was also found in TAM-related endometrial polyps (Hachisuga *et al*, 2003).

The purpose of the present study was to investigate any differences in the frequency of mutations in codon 12 of the K-ras gene in endometrial samples of patients treated with adjuvant TAM or TOR. We also correlated the presence of K-ras mutations with the duration of TAM treatment as well as ultrasonographically confirmed increased thickness of the endometrium under different conditions of menstruation.

## MATERIALS AND METHODS

### Patients

Since April 1993, gynaecologic examinations of patients who received adjuvant hormonal treatment after undergoing surgery for breast cancers have been performed at the outpatient gynaecologic clinic of the Fukuoka University Hospital. All patients underwent a pelvic examination and Papanicolau cervical smear tests followed by transvaginal ultrasonography (TVU), using an Aloka 3500 sector scanner with a 5.0-MHz transvaginal transducer (Aloka, Tokyo, Japan). The endometrial thickness was recorded by measuring the double layer at the widest points anteroposterior across the uterine cavity. An endometrial thickness of less than 5 mm is defined as normal in postmenopausal women. Between February 2003 and September 2004, the examinations for K-ras mutation in the endometrium were offered 117 breast cancer patients, who had received adjuvant hormonal treatment. Four patients refused to undergo examination for K-ras mutations. Although we tried to obtain endometrial cytologic samples from all of the remaining 113 patients, it was not possible in six patients due to cervical stenosis. As a result, 107 patients, including 86 with TAM treatment and 21 patients with TOR treatment, underwent examinations for the presence of K-ras mutations. In total, 20 patients underwent anthracycline or taxane based chemotherapy. In total, 15 patients underwent adjuvant radiotherapy for the prevention of the local recurrence of breast cancer. No patient underwent radiotherapy for castration of ovarian function or received hormonal adjuvant therapy except TAM, TAM and gonadotrophin-releasing hormone analogue (GnRHa) or TOR. In addition, no patients demonstrated abnormal cervical smear test findings in this study.

### Specimen sampling

After a cervical smear test was performed, the discharge around the uterine cervix was cleaned away to avoid any contamination of the uterine cervical cells in the cytologic samples of the endometrium. Fresh endometrial cytological samples were obtained using an endocyte device (Laboratorie CCD, Paris, France). The cellular material was divided into two parts: one was smeared onto glass slides and processed for routine Papanicolaou staining while the other was used for an analysis of a mutation in codon 12 of the K-ras gene. All endometrial smears in this study were microscopically confirmed by cytopathologists to exhibit aggregates of the endometrial glandular and stromal cells.

### Detection of mutations in codon 12 of the K-ras gene

DNA was extracted from cytological specimens using the standard phenol and chloroform method. Mutations in codon 12 of the K-ras gene were analysed by an enriched polymerase chain reaction (PCR)-enzyme-linked minisequence assay (ELMA) (Sumitomo Metal Industry, Inc., Tokyo, Japan) (Matsubayashi *et al*, 1999). This assay was based on the enrichment of the mutant K-ras gene as previously described (Levi *et al*, 1991), followed by incorporating a biotin-labelled nucleotide specific for the mutant gene, which was then quantified enzymatically with a chromogenic substance. The PCR amplified K-ras gene was captured by the probes that were designed to detect the K-ras codon 12 wild type (GGT) and six mutants (GAT, GCT, GTT, AGT, CGT, TGT), which were ultimately measured using a microtitre plate reader for detection and quantification. The results of semiquantitative analysis were scored as (3+), (2+), (1+), (+−) and (−), according to the percentage of the mutant ras genes. Approximately (3+), (2+), (1+), (+−) and (−) represented more than 20, 2–20, 0.2–2, less than 0.2% and none (not detected) of the mutant, according to the manufacturer’s instructions. Quantitative analysis of K-ras mutations is reported to provide a useful tool for diagnosing pancreatic cancer when the percentage of K-ras mutations is high (Tada *et al*, 2002a). We quantitatively divided the presence of K-ras mutations into two groups: low (+− and +) and high (2+ and 3+).

The oligonucleotide primers were as follows: (1) upstream for the first and second PCR, 5′-TAAACTTGTGGTAGTTGGAACT-3′; (2) downstream for the first PCR, 5′-GTTGGATCATATTCGTACAC-3′ and (3) downstream for the second PCR, 5′-CAAATGATCTGAATTAGCTG-3′. In total, 1 *μ*l of a 10-fold dilution of the first PCR product was digested with 2.5 U of the restriction enzyme *Bsr*I (New England Biolabs, Beverly, MA, USA). The detailed method has been described in previous studies (Matsubayashi *et al*, 1999; Hachisuga *et al*, 2003).

This study (no. 158) was approved by the ethics committee of Fukuoka University School of Medicine. Informed content was obtained from each patient.

### Statistical analysis

The *χ*^2^ test was used to assess the association between categoric variables. Statistical significance was considered to exist at a value of *P*<0.05.

## RESULTS

### Analyses of patients undergoing TAM treatment

#### Clinical findings

The age of the patients undergoing TAM treatment ranged from 27 to 71 years with a mean of 50.5 years. The patients were treated with 20 mg of TAM daily for 3 to 121 months with a mean of 36.1 months (the TAM dose ranged from 1.8 to 72.6 g). When examining for K-ras mutations, 24 patients were found to have regular, irregular or oligomenorrheic menstrual cycle and were classified as the premenopausal group. Five TAM-related amenorrheic patients, five chemotherapy and TAM-related amenorrheic patients and 15 Gn-RHa and TAM-related amenorrheic patients were included into the amenorrheic group. In total, 37 patients who had experienced menopause before TAM treatment were classified as the postmenopausal group. The endometrial thickness ultrasonographically ranged from 0.2 to 2.5 cm with a mean of 0.8 cm. In total, 25 (67.6%) of 37 postmenopsual patients showed an endometrial thickness of over 0.5 cm, while only three (12.0%) of 25 amenorrheic patients showed an endometrial thickness of over 0.5 cm (*P*<0.001).

#### Histopathologic findings

Histopathologic examination was performed in 35 patients with vaginal bleeding and/or recommendation of the histopathologic examination from cytopathologists, regardless of results of the K-ras mutation. The histopathologic diagnoses were comprised of 10 atrophic endometrial samples, six endometrial samples in the proliferative phase, three endometrial samples in the secretory phase, 14 endometrial polyps and two endometrial cancers. Of 51 TAM-treated patients without histologic examination, 47 are being followed more than 1 year after examination for K-ras mutation in our 6-month-interval gynaecologic screening program. Endometrial cancer has not been found in these patients.

#### K-ras mutations

**Figure 1:**
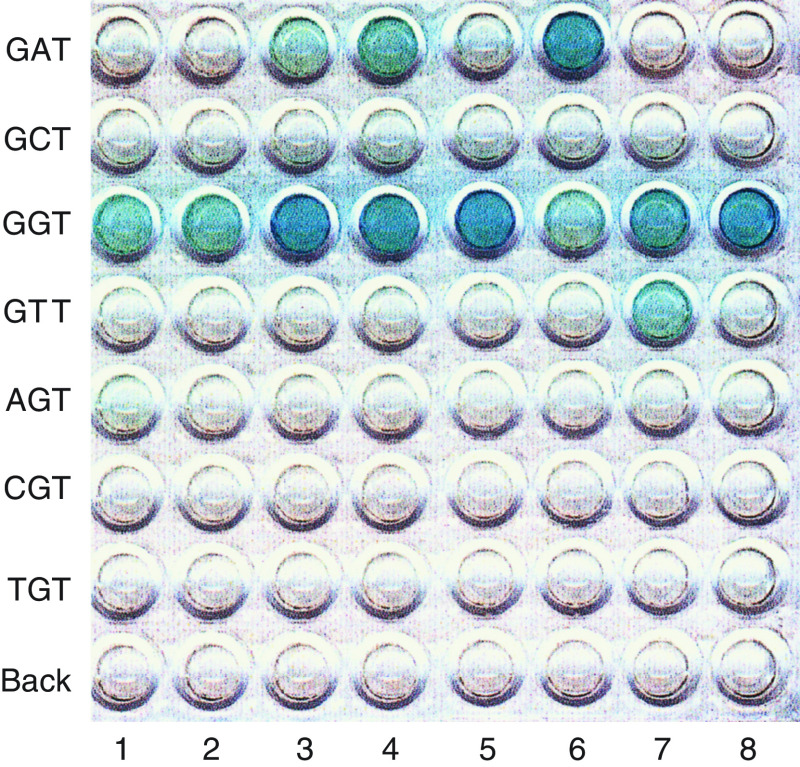
Semiquantitative analysis of mutations in codon 12 of K-ras using the enriched polymerase chain reaction (PCR)-enzyme-linked minisequence assay (ELMA; Sumitomo Metal Industry, Tokyo, Japan). Lanes 1and 2: toremifene-treated endometrium; lanes 3–8: tamoxifen-treated endometrium. The mutation types as determined by PCR-ELMA for lanes 1, 2, 5 and 8 were GGT (wild type), lane 3 was GAT(+−), lane 4 was GAT (1+), lane 6 was GAT (3+) and lane 7 was GTT (1+). BACK: background.

K-ras mutations were found in 35 (40.7%) of 86 patients undergoing TAM treatment (Table 1, Figure 1). Low levels (+− or +) and high levels (2+ or 3+) of K-ras mutation were found in 22 and 13 patients undergoing TAM treatment, respectively. Two patients exhibited multiple K-ras mutations (GTT of 2+ and GAT of 2+, and GCT of 3+ and GAT of 3+, respectively). In total, 54 patients were examined for K-ras mutation during TAM treatment and 32 patients after the cessation of the TAM treatment. Seven (29.1%) of 24 premenopausal patients, 18 (48.6%) of 37 postmenopasual patients and 10 (40.0%) of 25 amenorrheic patients were positive for K-ras mutation. Table 2 shows the correlation between the duration of TAM treatment and K-ras mutation in breast cancer patients during and after cessation of TAM treatment. The positive rate of K-ras mutation in the premenopausal group did not exhibit a significant difference from that of the K-ras mutation in the postmenopausal-amenorrheic group (*P*=0.18). The positive rate of K-ras mutation did not exhibit any significant difference between patients during and after cessation of TAM treatment both in the premenopausal (*P*=0.13) and in postmenopausal-amenorrheic (*P*=0.39) groups. The high level of K-ras mutation also did not reveal a significant difference between patients during and after cessation of TAM treatment both in the premenopausal (*P*=0.25) and in postmenopausal-amenorrheic (*P*=0.19) groups. In the premenopausal group, K-ras mutation was seen in seven (43.8%) of 16 patients with less than 47 months of TAM treatment, while none was seen in eight patients with more than 48 months of TAM treatment (*P*<0.03). In the postmenopausal-amenorrheic group, K-ras mutation was found in three (15.8%) of 19 patients with less than 23 months of TAM treatment, while it was found in 16 (61.5%) of 26 patients with 24–47 months of TAM treatment and nine (52.9%) of 17 patients with more than 48 months of TAM treatment (less than 23 months *vs* more than 24 months, *P*=0.002).

The positive rates of K-ras mutation were 45.0, 31.8 and 41.6% in the TAM-treated patients with endometrial thicknesses of less than 0.5, 0.6–0.9 and more than 1.0 cm, respectively. There was no significant correlation between the endometrial thickness and the presence of K-ras mutation (Table 3). TAM-treated patients with vaginal bleeding showed a greater frequency of endometrial thickness of more than 1.0 cm than those without vaginal bleeding (Table 4, *P*<0.001). Among the patients with endometrial thickness of greater than 1.0 cm, the patients with vaginal bleeding were more frequently detected to have a high level of K-ras mutations than those without vaginal bleeding (*P*<0.02). The correlation between K-ras mutation and certain histopathologic features is shown in Table 5. The positive rate of K-ras mutation was not siginificantly different between benign conditions, including atrophy and endometrium in the proliferative and sectretory phases, and neoplastic tumors including both polyps and cancer (*P*=0.40).

Seven (38.9%) of 18 patients with chemotherapy and 28 (41.2%) of 68 patients without chemotherapy were positive for K-ras mutation (*P*=0.86). No K-ras mutation was found in the cytologic samples of the uterine cervix that were randomly selected from 10 patients with the K-ras mutation positive endometirum.

### Analyses of the patients undergoing TOR treatment

#### Clinical findings

The age of the patients ranged from 41 to 86 years with a mean of 64.8 years. In total, 20 were postmenopausal patients and one was a chemotherapy and TAM-related amenorrheic patient. A total of 18 patients were seen during and three patients after cessation of TAM treatment. The patients were treated with 40 mg of TOR daily for 3–65 months with a mean of 29.4 months (the dose of TOR ranged from 3.6–78.0 g). The endometrial thickness ultrasonographically ranged from 0.3 to 2.2 cm with a mean of 1.1 cm. In total, 19 patients were found to have an endometrial thickness of over 0.5 cm.

#### Histopathologic findings

Histopathologic examination was in seven patients with vaginal bleeding and/or on the recommendation by cytopathologists of a histopathologic examination, regardless of the results of the K-ras mutation status. The histopathologic diagnoses were comprised of four endometrial polyps, two atrophic endometrial samples and one endometrium in the proliferative phase.

#### K-ras mutations

In the 21 patients with TOR treatment, an 86-year-old woman treated with 23 months of TOR treatment was detected to have a low level (GAT; 1+) of K-ras mutation. She complained of vaginal bleeding and ultrasonograph revealed an endometrial thickness of 1.2 cm. Histologic diagnosis of the biopsy specimen was an endometrial polyp.

## DISCUSSION

New technological modalities such as the PCR have helped to improve the molecular detection of cancer. One study reported that K-ras mutations can be detected in DNA samples prepared from endometrial aspirate specimens using the mutant-enriched PCR technique (Al-Jehani *et al*, 1998). Nucleotide substitutions identical to those found in the K-ras genes of the corresponding tumor DNA samples were found in all endometrial aspirate DNA samples in this study (Al-Jehani *et al*, 1998). Recently, a kit to quantitatively detect K-ras mutations, combining enriched PCR and ELMA, has been developed. The successful detection of K-ras mutations from pancreatic juice of patients with pancreatic cancer has been reported using this kit (Tada *et al*, 2002b).

One of the technical problems with cytologic sampling from the uterine cavity using an endocyte device is the difficulty of completely avoiding any contamination by the uterine cervical cells. Therefore, a mutation in codon 12 of the K-ras gene was randomly examined in cervical cell samples from 10 patients with a K-ras mutation positive endometirum, and a mutation in codon 12 of the K-ras gene was not detected in any cervical cell samples.

The genes most commonly affected by sporadically acquired point mutations in various types of cancers are in the ras gene family. The patterns seen in point mutations in cancers are the results of the interactions of three factors: (1) generation of an altered DNA base or nucleotide; (2) faulty DNA repair and (3) the biologic consequences of induced mutations (Kelly and Littman, 2002). In this study, the positive rates of K-ras mutation did not exhibit any significant difference between the postmenopausal-amenorrheic and the premenopausal groups. No K-ras mutation was seen in premenopausal patients with more than 48 months of TAM treatment, while it was seen in 52.9% of the postmenopausal-amenorrheic patients with more than 48 months of TAM treatment. Although the effect of the cessation of TAM treatment on the presence of the K-ras mutation needs to be better evaluated in a large case study, these results do suggest that menstruation probably plays an important role in the removal of K-ras mutations from the endometrium.

An increased risk of endometrial cancer has been specifically described in postmenopausal women, and this risk increases as the duration of TAM treatment increases (Fisher *et al*, 1994; Rutqvist *et al*, 1995; Bergman *et al*, 2000). Recently, in a chemoprevention trial, endometrial cancers were reported in patients who were premenopausal at the start of TAM treatment and who became amenorrheic during long-term TAM treatment along with having low serum oestrogen levels (Chang *et al*, 1998). The duration-related distribution of the K-ras mutation in amenorrheic patients was almost the same as that of the K-ras mutation in postmenopausal patients. Postmenopausal-amenorrheic patients with less than 23 months of TAM treatment exhibited a significantly lower incidence of K-ras mutations than did those with more than 24 months of TAM treatment.

In a quantitative analysis of K-ras mutations, the high level of the K-ras mutation was not positively associated with neoplastic changes, but it is interesting that a high level of K-ras mutation was more frequently found in symptomatic patients with endometrial thickness of greater than 1.0 cm. A large case study will need to be undertaken to clarify this provocative finding.

One previous study showed that endometrial thickness significantly increased during the course of treatment with no differences between TAM and TOR (Tomas *et al*, 1995). The authors of this study suggest TOR to have an oestrogenic effect on the endometrium similar to that of TAM. This finding has been confirmed in this report. Of the 21 TOR-treated patients, 19 had an endometrial thickness of over 0.5 cm, but only one K-ras mutation was detected in these patients. There is no correlation between endometrial thickness and K-ras mutation in the TAM-treated patients.

In summary, K-ras mutation was found in 40.7% of TAM-treated endometrium samples, whereas K-ras mutation was found in one TOR-treated endometrium. TOR may have a lower potential genotoxicity than TAM. Although the effect of the cessation of TAM treatment on the presence of the K-ras mutation needs to be more thoroughly evaluated in a large case study, these results appear to suggest that menstruation plays an important role in the removal of K-ras mutations. The presence of the K-ras mutation is significantly influenced by the duration of TAM treatment, but it does not correlate with endometrial thickness as measured by ultrasonography.

## Figures and Tables

**Table 1:** Types of K-ras mutations in the 35 tamoxifen-treated patients

**Type**	**+−**	**1+**	**2+**	**3+**	**Total**
GAT	4	3	6	2	15
GCT	3	2	1	3	9
GTT	5	3	2	1	11
AGT	0	0	0	0	0
CGT	1	0	0	0	1
TGT	1	0	0	0	1

Total	14	8	9	6	37^a^

a Two patients showed multiple K-ras mutations (GTT of 2+ and GAT of 2+, and GCT of 3+ and GAT of 3+, respectively).

**Table 2:** Duration of tamoxifen treatment and K-ras mutation in breast cancer patients during tamoxifen treatment and after cessation of tamoxifen treatment

		**K-ras mutation during TAM treatment**					**K-ras mutation after cessation of TAM treatment**			
**Duration (months)**	**No. of cases**	**(−)**	**Low**	**High**	**Positive rate (%)**	**No. of cases**	**(−)**	**Low**	**High**	**Positive rate (%)**
*Premenopausal group*										
1–23	4	2	1	1	50.0	1	1	0	0	0.0
24–47	8	4	3	1	50.0	3	2	1	0	33.3
48−*	3	3	0	0	0.0	5	5	0	0	0.0

*Postmenopausal-amenorrheic group*										
1–23**	14	12	2	0	12.5	5	4	0	1	20.0
24–47	13	6	6	1	53.8	13	4	5	4	69.2
48–	12	5	3	4	58.3	5	3	1	1	40.0

Total	54	30	15	7		32	19	7	6	

* *P*<0.03 (less than 47 months *vs* more than 48 months).

** *P*=0.002 (less than 23 months *vs* more than 24 months).

**Table 3:** Endometrial thickness and K-ras mutation in tamoxifen-treated breast cancer patients

		**K-ras mutation**			
**Endometrial thickness (cm)**	**No. of cases**	**(−)**	**Low**	**High**	**Positive rate (%)**
*Premenopausal group*					
−0.5	6	3	2	1	50.0
0.6−0.9	12	10	1	1	16.7
1.0−	6	4	2	0	33.3

*Amenorrheic group*					
−0.5	22	12	7	3	45.5
0.6–0.9	1	1	0	0	0.0
1.0−	2	2	0	0	0.0

*Postmenopausal group*					
−0.5	12	7	3	2	41.7
0.6–0.9	9	4	3	2	55.6
1.0−	16	8	4	4	50.0

Total	86	51	22	13	40.7

**Table 4:** Endometrial thickness and K-ras mutaion in tamoxifen-treated breast cancer patients with and without vaginal bleeding

	**Pateints with vaginal bleeding**					**Patients without vaginal bleeding**				
		**K-ras mutation**					**K-ras mutation**			
**Endometrial thickness (cm)**	**No. of cases**	**(−)**	**Low**	**High**	**Positive rate (%)**	**No. of cases**	**(−)**	**Low**	**High**	**Positive rate (%)**
−0.5	2	1	0	1	50.0	32	15	12	5	53.1
0.6–0.9	1	1	0	0	0.0	27	20	4	3	25.9
1.0−	11	4	3	4*	63.6	13	10	3	0	23.1

Total	14	6	3	5	57.1	72	45	19	8	37.5

* *P*<0.02 (patients with vaginal bleeding *vs* patients without vaginal bleeding).

**Table 5:** Histopathologic features and K-ras mutation in the 35 tamoxifen-treated patients

		**K-ras mutation**			
**Endometrium**	**No. of cases**	−	**Low**	**High**	**Positive rate (%)**
Atrophy	10	4	4	2	60.0
Proliferative phase	6	2	4	0	66.7
Secretory phase	3	2	1	0	33.3
Polyp	14	8	1	5	42.9
Cancer	2	1	1	0	50.0

Total	35	17	11	7	51.4
